# A Near Fatal Sneeze Spontaneous Splenic Rupture: A Case Report and Review of the Literature

**DOI:** 10.5811/cpcem.2017.2.32847

**Published:** 2017-05-24

**Authors:** Gregory W. Reinhold, Tina K. Melonakos, Daniel T. Lyman

**Affiliations:** * Promedica Monroe Regional Hospital, Department of Emergency Medicine, Monroe, Michigan; † Promedica Monroe Regional Hospital, Department of Pharmacy, Monroe, Michigan

## Abstract

A 79-year-old female called 911 for abdominal pain in her left upper quadrant with radiation through to her back and left shoulder for three hours. Upon arrival to the emergency department her physical exam was positive only for tenderness in the left upper quadrant of her abdomen. The patient denied any history of trauma but reported she “did sneeze three times” just prior to the onset of her pain. Computed tomography angiography of the abdomen and pelvis was obtained to evaluate for vascular pathology. The radiologist immediately called with concern for splenic laceration. The general surgeon took the patient directly to the operating room where she underwent a splenectomy and recovered without sequelae. This is the first case report of spontaneous splenic rupture that resulted after the act of sneezing. It is important to be aware of this rare clinical entity because early recognition can be life saving.

## INTRODUCTION

Atraumatic splenic rupture (ASR) and spontaneous splenic rupture (SSR) are rare, life-threatening events, typically not at the top of a clinician’s differential for acute abdominal pain in the absence of trauma. The purpose of this paper is to bring this diagnosis to the forefront of clinicians’ minds, given the potential catastrophic sequelae if undiagnosed.

Historically, the first documented cases of ASR were by Rokitansky in 1861 and Atkinson in 1874.^1^ In 1927 Weidemann defined “spontaneous splenic rupture” as that resulting from an incident without external force. In 1966 Knoblish further differentiated “non-traumatic rupture of a pathological spleen” from the extremely rare “non-traumatic splenic rupture of unknown etiology.”^2^ In other words, this specified a true SSR as occurring in spleens without pathologic disease.

Etiology of ASR has largely been hypothesized to result from three primary mechanisms, the first being mechanical distention secondary to parenchymal infiltration associated with hematologic malignancy such as leukemia or lymphoma. The second is splenic infarct causing capsular hemorrhage and rupture, and the third is the existence of an underlying coagulopathy.^3^ It is important to note these mechanisms involve pathology of the spleen and thus would not be categorized as true SSRs.

Recent literature has found the incidence of true SSR varies from <1% to 7% with a mortality rate of approximately 12.2%.^4^,^5^ SSRs are graded using the following criteria put forth by Orloff and Peskin: first, no history of trauma; second, no evidence of other organ disease known to adversely affect the spleen; third, no evidence of perisplenic adhesions or scarring of the spleen suggestive of prior trauma; and fourth, aside from hemorrhage and rupture, the spleen should be normal on gross inspection and histologic examination.^6^ In 1991 Crate and Payne added a fifth criterion that full virological studies of acute and convalescent serum should show no significant rise in antibody titers.^7^

## CASE REPORT

Our case began with a 79-year-old female who called 911 from her home for transport to the emergency department (ED) for evaluation of abdominal pain in her left upper quadrant with radiation through to her back and left shoulder, which had been present for approximately three hours. The patient had a past medical history significant for hypertension and hypercholesterolemia for which she took lisinopril and rosuvastatin daily.

Upon arrival at the patient’s home, the emergency medical services (EMS) team reported that the patient initially appeared well with normal vital signs but then began to deteriorate. The patient’s blood pressure fell to 61/38 mmHg and she had a near syncopal episode prompting EMS to administer intravenous (IV) fluids and commence transport to the ED for further evaluation.

When the patient arrived to the ED she was awake and alert with mild distress from her pain but interacting appropriately. Her vital signs were blood pressure of 101/56 mmHg, pulse rate of 74 beats per minute and an oxygen saturation of 100% on room air. Her physical exam was positive for tenderness in the left upper quadrant of her abdomen, but it was soft with no guarding or rigidity. She showed no signs of focal neurologic deficit, jugular venous distension, heart murmurs or abnormal lung sounds. Her extremities had equal distal pulses with good capillary refill. Her medical history provided no identifiable explanation for her pain. The patient denied any history of trauma but reported she “did sneeze three times” just prior to the onset of her pain. Given the patient’s symptom of sudden onset abdominal pain with radiation to her back accompanied by hypotension, abdominal aortic aneurysm (AAA) was immediately considered. A bedside ultrasound was then performed but results were limited due to bowel gas present. Initial interpretation of her exam showed no obvious AAA, no definite free fluid and no evidence of pericardial tamponade. In the meantime vascular and general surgery were notified of the case, and since the patient’s blood pressure had remained stable the decision was made to obtain computed tomography (CT) angiography of the abdomen and pelvis to further evaluate for vascular pathology.

**Image 1:**
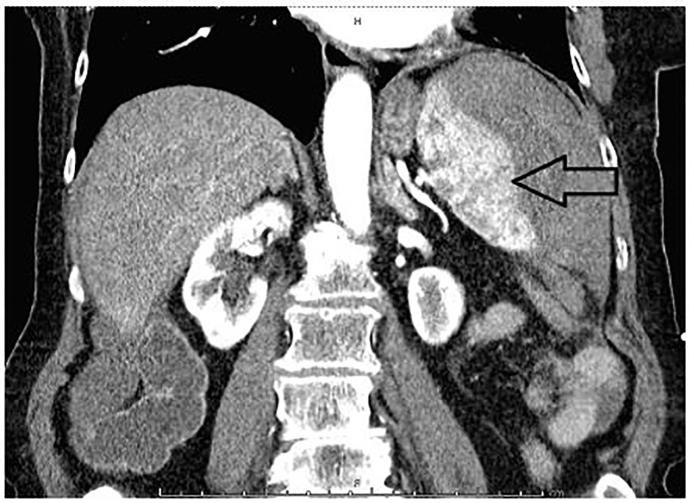
Computed tomography of abdomen and pelvis in coronal view showing splenic laceration.

Following the CT, the radiologist immediately called with preliminary results concerning for splenic laceration and hematoma with hemorrhagic abdominal and pelvic ascites (Image 1). After arriving back in the ED, the patient’s blood pressure decreased to 68/45 mmHg. She was given a one-liter IV fluid bolus and was transfused one unit of packed red blood cells. The CT results and clinical deterioration were communicated to the general surgeon who took the patient directly to the operating room where she was found to have a ruptured spleen in multiple pieces with a large amount of free intraperitoneal blood. A splenectomy was performed and the patient recovered without sequelae. The pathology report revealed splenic fracture with otherwise-normal splenic tissue. Lab results showed the patient’s initial liver functions and hemoglobin were within normal limits and a mononucleosis screen was negative.

CPC-EM Capsule

What do we already know about this clinical entity?

Spontaneous splenic rupture is a rare clinical entity and there has never been a case report documenting it as the result of sneezing.

What makes this presentation of disease reportable?

Splenic rupture is not typically in the differential for non-traumatic abdominal pain. This case illustrates why it should be considered even without history of trauma.

What is the major learning point?

Trauma is not required to have splenic rupture.

How might this improve emergency medicine practice?

Clinicians who keep this case in mind are more likely to identify spontaneous splenic rupture in the absence of trauma.

During her recovery the patient was questioned concerning any sustained trauma or recent illness. She continued to deny any obvious inciting event but recalled she had three forceful sneezing episodes prior to the onset of her pain. From a clinical standpoint her sneezing episodes were the only identifiable trigger for this patient’s SSR.

## DISCUSSION

SSR is a rare entity defined by the rupture of the spleen without antecedent injury. Most of the literature related to SSR is described in case reports. The term *spontaneous rupture* is often misleading. Wright and Prigot stated: “There is no such clinical entity as spontaneous rupture of the normal spleen.”^9^ Johnson suggested that careful questioning will always elicit a history of injury.^10^

The pathologic features of SSR of a normal spleen are similar to those with a history of trauma. The main findings on macroscopic examination are a subscapular hematoma, tears into the parenchyma, laceration of the pedicle, fragmentation and perisplenic hematoma.^11^ The microscopic examination of the spleen is different between the two groups. Farhi and Ashfaq found that in traumatic splenic ruptures there was an increase in the germinal center proliferation and of marginal-zone hyperplasia compared to the non-traumatic spleens of corpses.^12^

Many theories have been proposed to explain spontaneous rupture of the spleen (Table 1). It has been postulated that the incidence of splenic rupture correlates with the size of the spleen, but in a retrospective study by Bauer et al., the percentage of normal-sized spleens that ruptured was as much as 48%.^13^ The primary risk factors are splenic infiltration by hematologic disease, splenic infarct, male gender, adulthood, and severe splenomegaly.^14^

The diagnosis of SSR is in fact a diagnosis of exclusion. It is not considered to be a primary diagnosis in the evaluation of abdominal pain with hypotension in the absence of trauma. The most common presenting symptom is left upper quadrant pain and it is often associated with orthostatic symptoms. Two signs are particularly suggestive of splenic rupture: Kehr’s sign (left diaphragmatic irritation resulting in referred pain to the left shoulder), and Balance’s sign (palpable tender mass in the left upper quadrant). Classically, patients are found to be in hypovolemic shock with signs of peritonitis on exam. For our patient the exact diagnosis was not made until further imaging studies had been done.

The management of splenic ruptures is based upon a grading scale from low to high depending on whether the splenic capsule is intact or ruptured.^3^ Low-grade splenic injuries may be managed conservatively as long as patients are hemodynamically stable. High-grade injuries typically require prompt surgical splenectomy to stabilize the patient.^8^

Confusing symptomatology arises for a couple of reasons. First, by virtue of the spleen’s position, local symptoms can be related to the left side of the chest, abdomen, and flank. Secondly, left-sided chest pain coupled with left shoulder pain and hemodynamic instability seen in splenic rupture can mimic myocardial infarction, pulmonary embolism or dissecting/ruptured aortic aneurysm. Finally, as a result of hemorrhage into the abdominal cavity, acute splenic rupture can masquerade as almost any cause of acute abdominal pain.

## CONCLUSION

This is the first documented case report of spontaneous splenic rupture that resulted after the act of sneezing. It was fortunate for our patient that the diagnosis was made quickly with the assistance of CT imaging and operative intervention was obtained in a timely manner. The patient recovered without sequelae. It is important to be aware of this rare clinical entity during practice because early recognition can be life saving.

## Figures and Tables

**Image 2:**
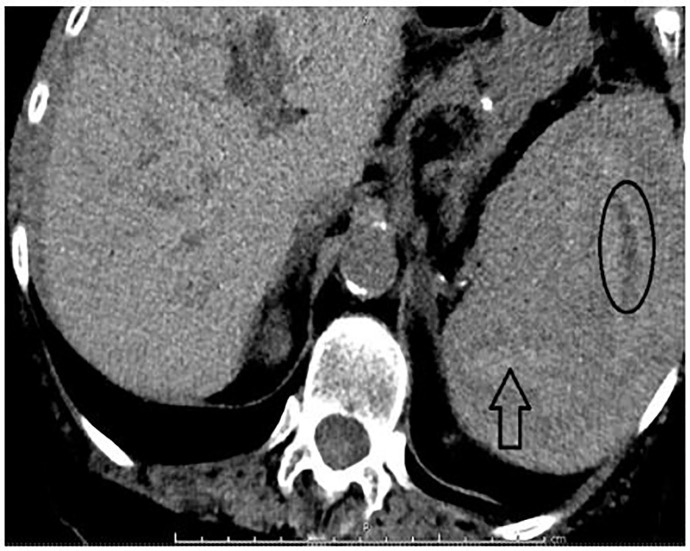
Computed tomography of abdomen and pelvis in transverse view showing multiple hypoechoic densities within splenic parenchyma suggestive of splenic laceration (arrow) and hematoma formation (oval).

**Table:** Theories proposed to explain the spontaneous rupture of the spleen. [1^–21]^

1.	Localized involvement of the spleen with a pathologic process where, upon rupture, all evidence of pathologic changes is destroyed.
2.	Acute splenic congestion secondary to splenic vein spasm.
3.	Splenic congestion secondary to chronic portal venous congestion.
4.	Excessively mobile spleen causing mechanical obstruction and rupture.
5.	Splenic artery aneurysm or degeneration.
6.	Minor trauma that went overlooked.
7.	Sudden elevation in abdominal pressure (i.e. a heavy meal, defecation, lifting, sexual intercourse, coughing, vomiting, pregnancy, colonoscopy, ERCP ).

*ERCP,* endoscopic retrograde cholangiopancreatography.

## References

[1] Rokitansky KF (1861). Two recent cases of spontaneous rupture of the spleen. Wochenblatt der Zeitschrift der k Gesellschaft der Aerzte in Wien.

[2] Lieberman ME, Levitt MA (1989). Spontaneous rupture of the spleen: A case report and literature review. Am J Emerg Med.

[3] Maqbool MS, Brush RG, Northrup M (2014). Atraumatic splenic rupture. Ultrasound Q.

[4] Renzulli P, Hostettler A, Schoepfer AM (2009). Systematic review of atraumatic splenic rupture. Br J Surg.

[5] Laseter T, McReynolds T (2004). Spontaneous splenic rupture. Mil Med.

[6] Orloff MJ, Peskin GW (1958). Spontaneous rupture of the normal spleen; a surgical enigma. Int Abstr Surg.

[7] Crate ID, Payne MJ (1991). Is the diagnosis of spontaneous rupture of a normal spleen valid?. J R Army Med Corps.

[8] Weaver H, Kumar V, Spencer K (2013). Spontaneous splenic rupture: A rare life-threatening condition; Diagnosed early and managed successfully. Am J Case Rep.

[9] Wright LT, Prigot A (1939). A traumatic subcutaneous rupture of the normal spleen. Arch Surg.

[10] Johnson N (1954). Traumatic rupture of the spleen; a review of eighty-five cases. Aust N Z J Surg.

[11] Toubia NT, Tawk MM, Potts RM (2005). Cough and spontaneous rupture of a normal spleen. Chest.

[12] Farhi DC, Ashfaq R (1996). Splenic pathology after traumatic injury. Am J Clin Pathol.

[13] Bauer TW, Haskins GE, Armitage JO (1981). Splenic rupture in patients with hematologic malignancies. Cancer.

[14] Giagounidis AA, Burk M, Meckenstock G (1996). Pathologic rupture of the spleen in hematologic malignancies: two additional cases. Ann Hematol.

[15] Aubrey-Bassler FK, Sowers N (2012). 613 cases of splenic rupture without risk factors or previously diagnosed disease: a systematic review. BMC Emerg Med.

[16] Wehbe E, Raffi S, Osborne D (2008). Spontaneous splenic rupture precipitated by cough: A case report and a review of the literature. Scand J Gastroenterol.

[17] Kocael PC, Simsek O, Bilgin IA (2014). Characteristics of patients with spontaneous splenic rupture. Int Surg.

[18] Randriamarolahy A, Cucchi JM, Brunner P (2010). Two rare cases of spontaneous splenic rupture. Clin Imaging.

[19] Wang C, Tu X, Li S (2011). Spontaneous rupture of the spleen: a rare but serious case of acute abdominal pain in pregnancy. J Emerg Med.

[20] Anyfantakis D, Kastanakis M, Karona P (2014). Spontaneous rupture of the spleen masquerading as a pulmonary infection. Case Rep Surg.

